# Analytical and Antimicrobial Characterization of Zn-Modified Clays Embedding Thymol or Carvacrol

**DOI:** 10.3390/molecules29153607

**Published:** 2024-07-30

**Authors:** Loris Pinto, Federico Baruzzi, Roberto Terzano, Francesco Busto, Alessia Marzulli, Carmela Magno, Stefania Cometa, Elvira De Giglio

**Affiliations:** 1 Institute of Sciences of Food Production (CNR-ISPA), Via G. Amendola 122/O, 70126 Bari, Italy; loris.pinto@ispa.cnr.it (L.P.); federico.baruzzi@ispa.cnr.it (F.B.); alessia.marzulli@ispa.cnr.it (A.M.); 2 Department of Soil, Plant and Food Sciences, University of Bari, Via Orabona 4, 70126 Bari, Italy; roberto.terzano@uniba.it; 3 Department of Chemistry, University of Bari, Via Orabona 4, 70126 Bari, Italy; francesco.busto@uniba.it; 4 Consorzio INSTM, Via Giusti 9, 50121 Firenze, Italy; 5 VIBAC SpA, Strada Ticineto Salita San Salvatore 40, 15040 Ticineto, Italy; carmela.magno@vibac.it; 6 Jaber Innovation s.r.l., Via Calcutta 8, 00144 Rome, Italy

**Keywords:** zinc-modified clays, carvacrol, thymol, natural antimicrobial compounds, analytical characterization

## Abstract

Carvacrol and thymol are broad-spectrum natural antimicrobial agents. To reduce their volatility and improve their antimicrobial performance, synergistic systems were prepared loading the active molecules in zinc-modified clays. Montmorillonite (MMT) and zeolite (ZEO) were modified with zinc ions (ZnMMT and ZnZEO), with well-known antimicrobial properties, and then with carvacrol or thymol, reaching the 26 ± 3% and 33 ± 2% *w*/*w* of loading, respectively. The resulting hybrid materials were characterized by FT-IR, XPS, XRD, TGA, and GC-MS to evaluate carvacrol/thymol release in simulating food matrices. Antimicrobial assays carried out using spoiler and pathogenic bacterial strains showed that the antimicrobial activity of both thymol and carvacrol was largely preserved once they were loaded into Zn-modified clays. However, MMT hybrids showed an antibacterial activity significantly higher than ZEO hybrids at 50 mg/mL of thymol and carvacrol. For this reason, deeper antimicrobial evaluations were carried out only for ZnMMT composites. ZnMMT loaded with thymol or carvacrol produced inhibition zones against most of the target strains, also at 3.12 mg/mL, while the positive controls represented by the single molecule thymol or carvacrol were not active. The hybrid materials can be useful for applications in which the antimicrobial activity of natural molecules need to be displayed over time as requested for the control of microbial pathogens and spoilage bacteria in different applications, such as active packaging, biomaterials, and medical devices.

## 1. Introduction

Essential oils (EOs) are a mixture of different compounds such as terpenes (e.g., p-cymene, limonene), terpenoids (e.g., thymol, carvacrol), and phenylpropenes (e.g., eugenol, vanillin), which may display antimicrobial activity [1]. Among them, thymol and carvacrol have shown antibacterial activity, by direct contact and in gaseous form, for applications in the food sector [2] and wound dressing [3]. These terpenoids have been included in different biopolymers for the development of active packaging [4], and functional biomaterials [5,6]. However, as reviewed by Hajibonabi et al. [7], thymol and carvacrol have high volatility, low stability, and high hydrophobicity, which limit their application in several fields. In order to address these limitations, several nanocarriers (e.g., liposomes, polysaccharides, polymeric nanoparticles, and nanoemulsions) have been proposed as drug delivery systems of thymol and carvacrol [7]. The inclusion of essential oil compounds into nanoclays has also been suggested to achieve a controlled release over time and to protect EOs compounds from high temperatures, often employed during polymer processing [8].

Different preparation methods of clay hybrids including thymol or carvacrol such as direct mixing [9], mechanical milling [10], and supercritical solvent impregnation [11] have been proposed. Recently, Cometa et al. [12] described the preparation of zeolite–thymol composite material using a dry and solvent-free approach along with the analytical and antibacterial performances of the clay hybrid.

With regard to the clay material used to include thymol or carvacrol, montmorillonite is widely employed [13,14], although other clays have also been assayed [9,10,15]. Zeolite frameworks are also used for the organic functionalization with carvacrol and thymol [11,12].

In recent years, several applications of zeolite hybrids with thymol have been proposed in the fields of active packaging and functional biomaterials. Giannakas et al. [16] developed an alginate/zeolite–thymol film with bacteriostatic activity towards *Staphylococcus aureus* on cheese, whereas a thymol–zeolite/low-density polyethylene film controlled the lipid oxidation in pork fillets [17]. A chitosan/polyvinyl alcohol film loaded with thymol/zeolite hybrid showed enhanced antibacterial activity against *Escherichia coli*, *Sta. aureus*, *Listeria monocytogenes*, and *Salmonella enterica* [18].

The antimicrobial use of metal ions, such as silver, copper, zinc, gold, platinum, and gallium, in their free or complexed form, has longstanding historical precedent [19].

As recently reported [20], the employ of metal oxide nanomaterials, such as ZnO, Fe_2_O_3_, TiO_2_, Ag_2_O, CaO, MgO, and CuO, as antimicrobial agents is extensively studied due to their low-toxicity, high stability, good biological properties, and relatively low cost.

In the case of ZnO, often employed as a nanoparticle (NP) structured material, the broad scientific literature sustains that its antimicrobial activity is produced by Zn^2+^ ions releasing. However, the mechanism of ZnO-NP antibacterial activity includes several pathways such as the reactive oxygen species (ROS) generation, the release of free Zn^2+^ ions, the contact with bacterial cell wall via electrostatic interaction followed by membrane disruption, internalization of ZnO-NP in bacterial cell, and consequent disorder and leakage [21].

Metal oxides and salts were also loaded into clays to produce hybrids with enhanced antimicrobial activity. For example, Zn absorption or loading in different clays was widely reported [22,23], also for the development of antimicrobial hybrids [24,25].

The capability of clays to exchange ions was exploited to produce hybrids with enhanced antimicrobial activity also when coupled with natural organic compounds. For example, Ag^+^/Zn^2+^ permutite/clove essential oil showed stronger antifungal efficacy than Ag^+^/Zn^2+^ permutite [26]. Tea tree oil-loaded chitosan/Zn^2+^ montmorillonite showed higher inhibition zones than tea tree oil-loaded chitosan/montmorillonite against *E. coli* [27].

To the best of our knowledge, there are no data comparing the antimicrobial activity of Zn-modified clays loaded with thymol or carvacrol. The aim of this study was the development and characterization of Zn-modified montmorillonite and zeolite loaded with thymol or carvacrol. The composites were characterized for their physicochemical properties and antibacterial effect against bacterial pathogens. Fourier transform infrared spectroscopy (FT-IR), X-ray diffraction (XRD), thermogravimetric analysis (TGA), X-ray photoelectron spectroscopy (XPS), and gas chromatography coupled with mass spectrometry (GC-MS) allowed for characterizing the bioactive composites. Zinc release and antioxidant activity were also evaluated.

The composites were evaluated against a group of target bacterial strains including the most important foodborne spoilers and pathogens, showing ZnMMT–thymol as the most active composite.

## 2. Results and Discussion

### 2.1. Chemical–Physical Characterization of the Zn-Modified Clays

**Figure 1:**
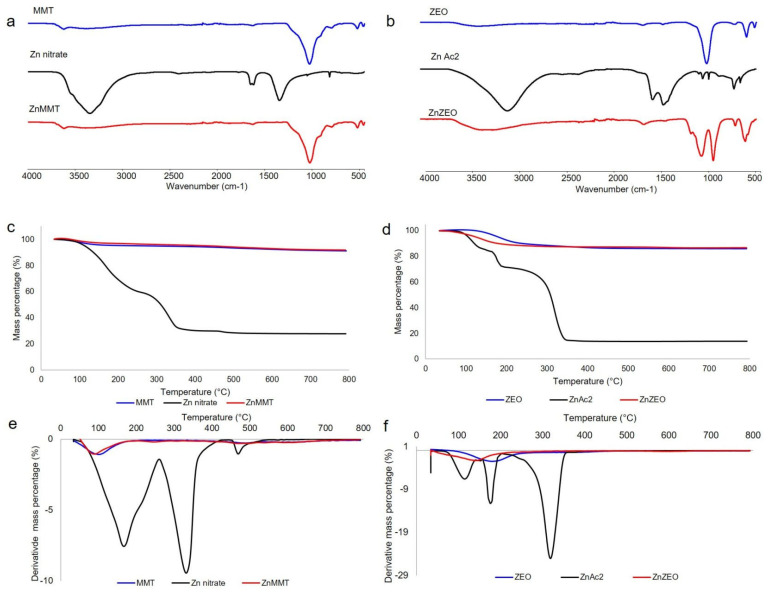
FT-IR/ATR (**a**), TGA (**c**), and DTGA (**e**) of ZnMMT and its precursors; FT-IR/ATR (**b**), TGA (**d**), and DTGA (**f**) of ZnZEO and its precursors.

In Figure 1a, the FT-IR/ATR spectra of ZnMMT and its precursors is reported. It is worth noting that the ATR peaks of ZnMMT were almost similar to those of MMT, i.e., a band at ~3620 cm^−1^ attributed to Al-OH group stretching and a band at 1028 cm^−1^, attributed to stretching vibration of Si-O [28]. No trace of zinc nitrate was detected in the ZnMMT sample. Thermogravimetric analysis, TGA and DTGA, reported in Figure 1c and 1e, respectively, also evidenced a strong similarity between MMT and ZnMMT clays. The residue of ZnMMT at 800 °C was 89.6% and the first thermal event, centered at 76 °C and corresponding to 4% weight loss, was due the loss of interlayer water, while the second event at 491 °C could be linked to the loss of coordination water, with a weight loss equal to 6.3%. ATR spectra of ZnZEO and its precursors were reported in Figure 1b. ATR of zinc acetate showed the band of OH stretching at 3075 cm^−1^ and the asymmetric and symmetric stretching of COO^−^, falling at 1547 and 1435 cm^−1^. Moreover, ATR analysis of ZnZEO evidenced that the calcination allowed for the almost total acetate removal. The band at 976 cm^−1^ present in ZEO spectrum was related to the Si–O–Si or Si–O–Al vibrations in tetrahedra or alumino- and silicon–oxygen bridges [12]. In ZnZEO system, in addition to the typical absorptions of clay, the analysis evidenced the presence of bands at about 3350 cm^−1^, which could be relate to the stretching vibration of OH present in Zn(OH)_2_ species. Other modes originating from this compound were observed at lower wavenumbers (in the range 1100–900 cm^−1^) [29]. TGA and DTGA of ZnZEO and its precursors are reported in Figure 1d and Figure 1f, respectively. Zinc acetate thermogravimetric analysis showed a first event linked to the presence of physically adsorbed water at about 115 °C (weight loss of 14%), a second event probably linked to coordination water at 175 °C (weight loss of 14%), and a third event at 278 °C (weight loss of 57%), ascribable to the transformation of anhydrous zinc acetate to ZnO, a process occurring at about 300 °C [30]. As far as the ZEO sample is concerned, the first event at 182 °C was relevant to desorption of physically adsorbed water or other volatiles within the zeolite cages, as well as the water located in zeolite cavities and bound to the non-framework cations (weight loss of 10.7%). The second event, at about 348 °C (weight loss 3.4%), could be associated to the water loss from hydration complexes formed with the exchangeable cations [31]. TGA (and DTGA) traces of the Zn-modified clay evidenced an anticipation of the first event, falling at about 141 °C, probably linked to the presence of Zn(OH)_2_, in agreement with what was observed by XRD analysis. The second event disappeared, while a third event, not present in the ZEO thermogram, fell at about 594 °C. In the literature, a third weight loss in the range (450–500 °C) for zeolites was reported to be due to the structural water and the destruction of the zeolite structure [32].

**Figure 2:**
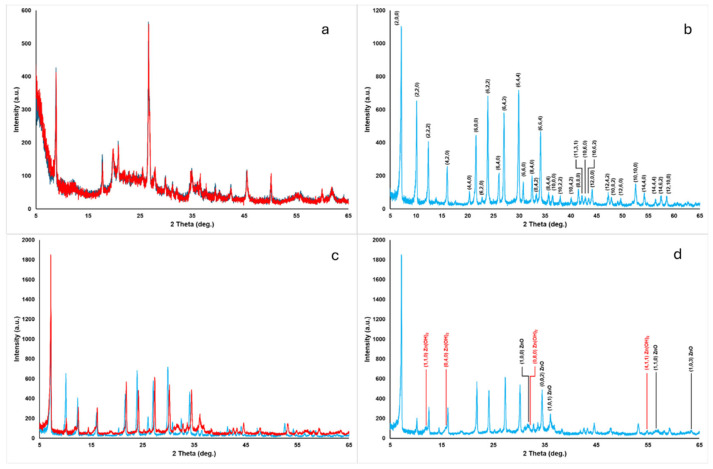
XRD patterns of (**a**) MMT (blue line) and ZnMMT (red line); (**b**) ZEO, with main diffraction peaks indexed; (**c**) ZEO (blue line) and ZnZEO (red line); and (**d**) ZnZEO, with main diffraction peaks of Zn(OH)_2_ and ZnO indexed.

As far as XRD analyses are concerned, MMT and ZnMMT showed the same XRD pattern (Figure 2a), suggesting a simple cation exchange process without structural modifications of the clay mineral. In Figure 2b, the XRD pattern of the zeolite 4A used in the experiments is reported together with the indexes of the main diffraction peaks, according to the reference 00-043-0142 PDF file (International Centre for Diffraction Data, https://www.icdd.com/, accessed on 30 June 2024). Simple calcination at 500 °C did not modify the zeolite structure while the Zn-modified zeolite (ZnZEO) presented several differences compared to the initial zeolite 4A (Figure 2c). Specifically, changes in the relative intensity of almost all the zeolite 4A diffraction peaks were observed together with a shift of all the peaks to higher 2Θ° values. The shift was confirmed adding a corundum internal standard. Alswatha et al. [33] observed the same modification of the diffraction pattern, suggesting a small compression of the zeolite crystalline structure after the treatment used to modify the zeolite with Zn (ZnZEO), without changing the cubic structure. New peaks were observed after the treatment of the zeolite with Zn at 11.98, 15.78, 31.39, 32.00, 35.83, and 54.64 2Θ° (Figure 2d) and were attributed to Zn(OH)_2_ (PDF n°00-020-1437) and ZnO (PDF n°01-079-0205), respectively.

XPS analysis proved to be particularly useful for understanding the changes in the clays surface composition. Unfortunately, composites containing thymol or carvacrol cannot be examined by XPS due to the volatility of these organic molecules. The atomic percentages of the elements detected on the surfaces of the investigated samples are reported in Table 1. Comparing XPS data relevant to MMT and ZnMMT samples, it can be observed the disappearance of the calcium signal in ZnMMT obtained with the Zn exchange procedure suggesting that calcium ions were replaced with zinc ones. The atomic percentages relevant to C1s, O1s, Al2p, Si2p, and Mg1s signals remained substantially unchanged in MMT and ZnMMT samples. No nitrogen signal relevant to Zn(NO_3_)_2_ reagent was detected on ZnMMT. As far as ZEO samples are concerned, XPS analysis suggests that the introduction of zinc can be associated both to the exchange with sodium ions and to an increase of the O1s/Si2p ratio, due to the ZnO and Zn(OH)_2_ species, revealed also by XRD analysis. Moreover, the ZEO-Zn(Ac)_2_ calcination at 500 °C induced a decrease of the C/Zn ratio from 1.6 to 1.2, indicating the removal of acetate molecules in agreement with what observed by ATR-FTIR analysis.

**Figure 3:**
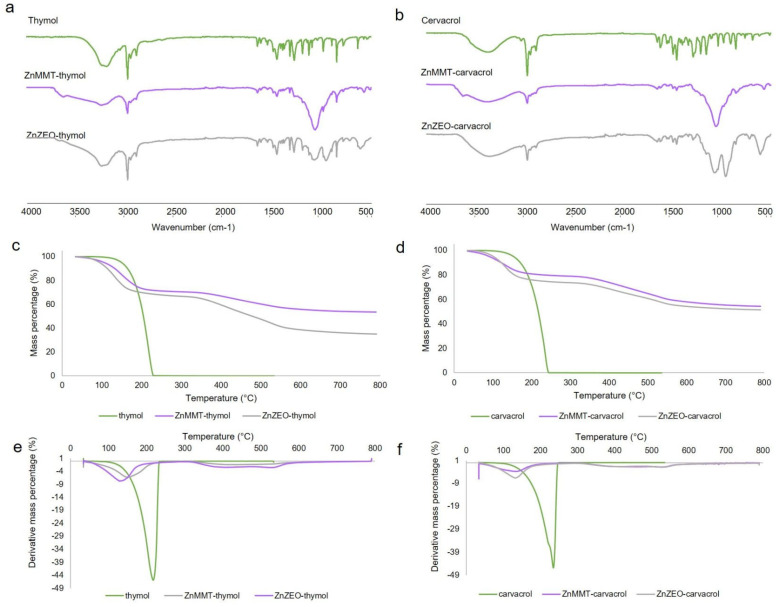
FT-IR/ATR (**a**), TGA (**c**), and DTGA (**e**) of thymol-based clays; FT-IR/ATR (**b**), TGA (**d**), and DTGA (**f**) of carvacrol-based clays.

FT-IR/ATR spectra of thymol and carvacrol, reported in Figure 3a,b, showed a band at about 3200 and 3360 cm^−1^, corresponding to phenolic O–H stretching involved in H bonds. The stretching of C–H fell in the 3000–2850 cm^−1^ range. The C=C stretching (1622 cm^−1^), –OH bending (1360 and 1362 cm^−1^), and C–O stretching (1242 and 1250 cm^−1^) were typical of phenolic groups of thymol [34] and carvacrol [35]. Moreover, a distinction between the two regioisomer spectra can be made considering the fingerprint region: the meta-substitution (thymol) brings the aromatic C–H bending absorption at 1058 cm^−1^, while the orthosubstitution (carvacrol) brings it at 994 cm^−1^, as reported in the literature [36]. As far as the hybrid composites ATR analyses are concerned, no significant variations in the thymol and/or carvacrol and ZnMMT and/or ZnZEO IR spectra were detected. However, in ZnMMT-active molecule spectra, a broadening of the Al–OH stretching band characteristic of MMT-based clay was observed. This can be related to the H-bonds instauration between the clay and the active molecules, as hypothesized in the discussion of TGA results (see below) and as previously reported by Essifi et al. [13].

As far as TGA analyses are concerned (Figure 3c,d), thymol and carvacrol compounds showed a decomposition peak centered at 183 °C [12] and 230 °C [37], respectively. Concerning the hybrid composites prepared (i.e., ZnMMT–thymol, ZnMMT–carvacrol, ZnZEO–thymol, and ZnZEO–carvacrol), two main mass losses were detected, i.e., a first weight loss below 220 °C (probably due to active molecule and/or water/volatiles evaporation) and a second weight loss between 300 and 600 °C. Similar results were also reported by Essifi et al. [13], which ascribed this second stage to the desorption of the active molecules interacting with the clay surface through hydrogen bonds between OH groups of thymol or carvacrol and OH groups of the hosting clay.

### 2.2. Antioxidant Activity Evaluation of Thymol and Carvacrol

**Figure 4:**
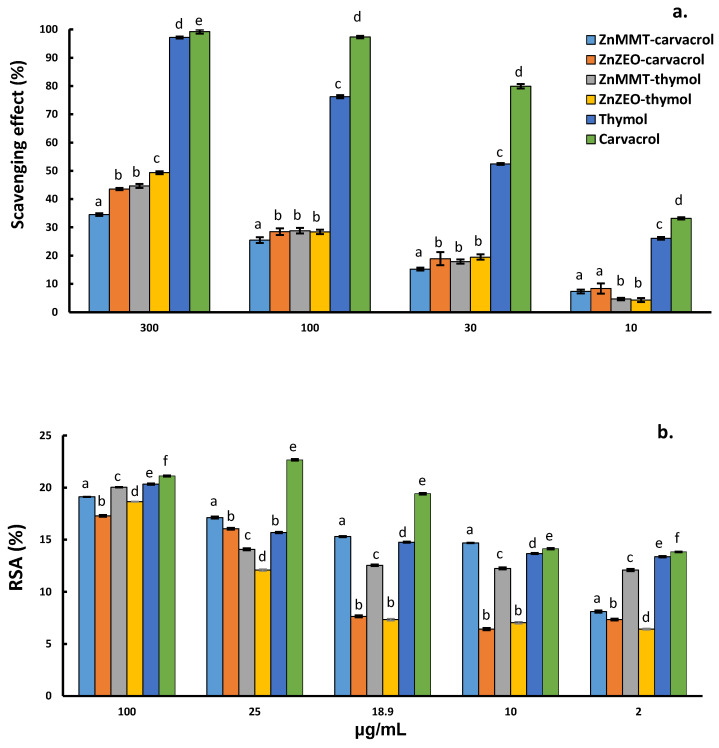
ABTS (**a**) and DPPH (**b**) assays for ZnMMT and ZnZEO loaded with thymol or carvacrol and free thymol and carvacrol. X-axis reports the concentration of carvacrol or thymol. One-way Student’s *t*-test analysis was applied to estimate differences between each organic molecule or composite materials in terms of antioxidant activity. Different lowercase letters indicate significant differences (*p* ≤ 0.05) among each concentration.

The antioxidant activity of thymol and carvacrol is well documented in the literature by different studies that describe the concentration-dependent scavenging effect [38,39,40]. The ABTS and DPPH antioxidant assays were performed on both thymol and carvacrol free molecules. As far as the ABTS test (Figure 4a), both molecules showed excellent antioxidant activity at maximum of the tested concentrations (300 μg/mL) with values of 97.2 ± 0.3% and 99.2 ± 0.7% for thymol and carvacrol, respectively. In this test, carvacrol provided a major antioxidant effect in the range 300–10 μg/mL in comparison to thymol, which shows a similar but less intense concentration-dependent trend. On the other hand, in the DPPH radical scavenging test (Figure 4b), samples provided a low antioxidant activity even at the higher concentration (100 μg/mL), with the highest RSA value (%) equal to 20.3 ± 0.1% and 21.1 ± 0.1% for thymol and carvacrol, respectively. For both molecules, a relationship between the percentages of the RSA values and the concentrations was observed, in agreement with previously reported data [38,39].

ABTS and DPPH tests have been also carried out on thymol and carvacrol loaded Zn-modified clays to verify if the molecules’ antioxidant activity can be modified by the presence of the clay matrix. In Figure 4, concentrations on X-axes are relevant to carvacrol or thymol loaded in the clays and evaluated by GC-MS.

ABTS test showed a decrease in antioxidant activity compared to free thymol and carvacrol, demonstrating the presence of a matrix effect.

The DPPH assay’s results are reported in Figure 4b. RSA% values for concentrations between 100 and 25 μg/mL remained substantially unchanged with respect to what observed for free molecules. On the other hand, at concentrations lower than 18.9 μg/mL, ZnMMT systems showed an almost stable antioxidant activity always better than ZnZEO systems.

### 2.3. Loading Capacity and Loading Efficiency of Thymol and Carvacrol by GC-MS Analysis

The optimized chromatographic procedure allowed for a quantitative evaluation of thymol or carvacrol showing an efficient separation of the components for an 8-min-long analysis (see Supplementary Materials, Figures S1 and S2).

Table 2 shows the four types of clays with their respective composites loaded with carvacrol or thymol. Measured concentrations, loading capacity (LC%), and loading efficiency (LE%) percentages are reported.

It can be observed that for all the composites, a loading efficiency percentage greater or proximal to 100% was detected, suggesting the effectiveness of the experimental procedure adopted. The LC% values are also in agreement with the experimental procedures adopted (see Section 3.3). Indeed, carvacrol-loaded systems were obtained using a physi-adsorption method with a clay/carvacrol ratio equal to 1:0.25 *w*/*w*. For thymol-loaded samples, a melting/crystallization cycle in the presence of the clay was performed using a ratio clay/thymol equal to 1:0.5 *w*/*w*.

**Figure 5:**
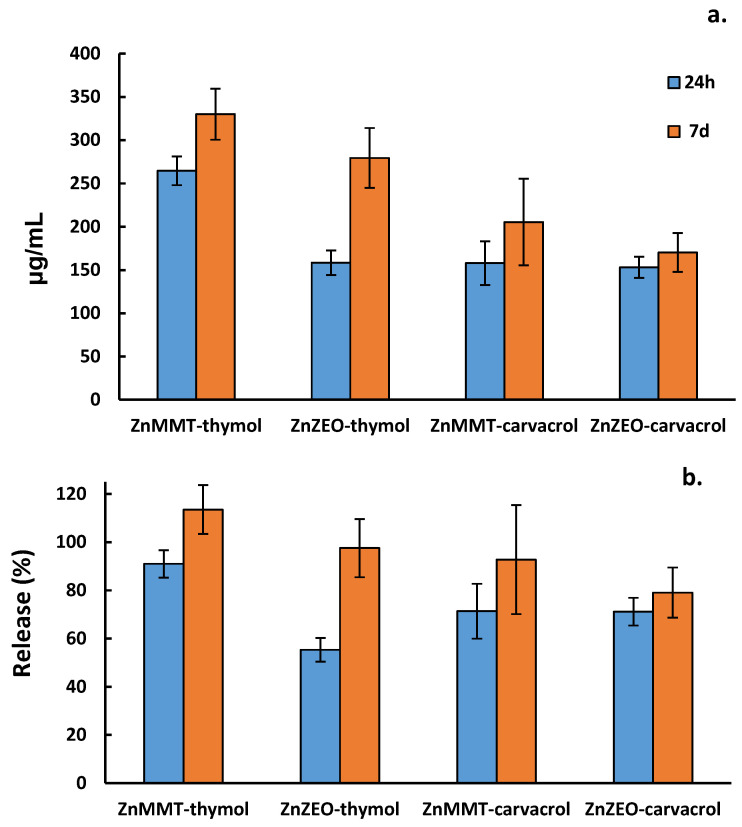
(**a**) Thymol or carvacrol amounts released in aqueous solution at 37 °C after 24 h and 7 days. (**b**) Thymol or carvacrol released percentages with respect to the initial loaded amounts.

GC-MS analysis was also employed to detect thymol or carvacrol released in aqueous solution from the loaded composites. The results are reported in Figure 5.

With regard to thymol, after 24 h, the ZnMMT system released a greater quantity than that observed for the ZnZEO system. On the other hand, carvacrol was released almost equally from the two systems. After 7 days all composites, except ZnZEO–carvacrol, released approximately the total loaded amount. These findings suggest that ZnMMT–thymol could be the most promising composite in terms of antimicrobial activity. This hypothesis, together with its zinc release ability, reported in Section 2.4, is really confirmed by the antimicrobial activity data (see Section 2.5). On the latter composite, quantification of thymol by GC-MS was performed after six months of ageing, observing a minimal thymol loss of approximately 2.5%.

### 2.4. Quantification of Zinc by Spectrophotometric Analysis

**Figure 6:**
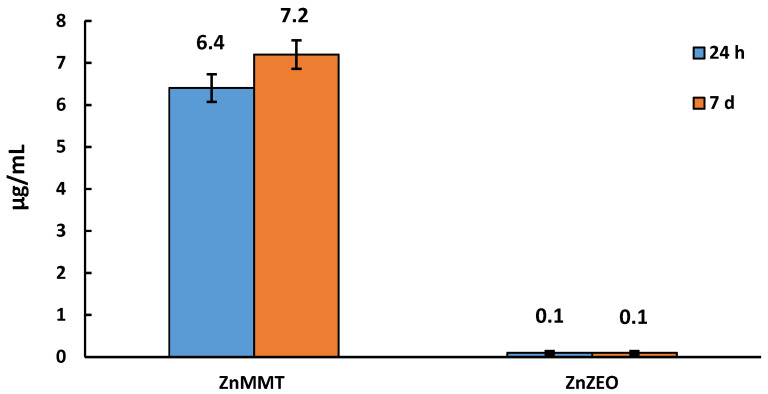
Zinc amount released from ZnMMT and ZnZEO in aqueous medium after 24 h and 7 days.

In Figure 6, the zinc amounts released from ZnMMT and ZnZEO in aqueous medium after 24 h and 7 days were reported. It can be observed that, using the same mass of the composites, the ZnMMT sample released more zinc than the ZnZEO one. Moreover, for the ZnZEO composite the low zinc amount released after 24 h did not increase after 7 days, while the ZnMMT sample showed a slight increase in release after 7 days. Despite to the lower surface atomic percentage of zinc recorded by XPS analysis, the ZnMMT composite showed a higher zinc release. This finding can be related to the presence of insoluble or slightly soluble species (ZnO and Zn(OH)_2_) detected in the ZnZEO composite.

**Figure 7:**
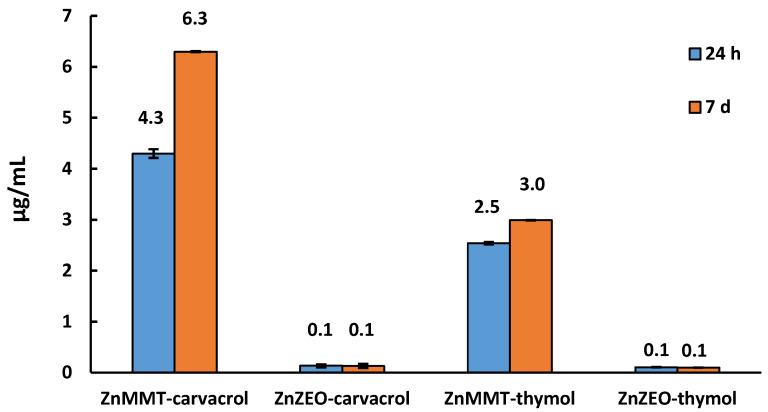
Zinc amount released from ZnMMT and ZnZEO, loaded with carvacrol or thymol, in aqueous medium after 24 h and 7 days.

Furthermore, zinc release tests were also carried out on the same Zn-modified clays after the addition of thymol or carvacrol, to understand whether the presence of the organic molecule can somehow modify the ionic release. Results were reported in Figure 7.

It can be confirmed that ZnMMT systems released more zinc than ZnZEO ones, as already observed in Figure 6. These data could support results reported for their antibacterial activity (see Section 2.5). ZnZEO-based composites released in both cases a very low amount of zinc, comparable with the quantity observed for the system without organic molecules. However, in the case of the ZnMMT system, we detected a lower amount of zinc released, and at the same tested composite mass, compared to the composite without thymol or carvacrol, as expected. Furthermore, the carvacrol-enriched ZnMMT system released more zinc than the thymol-enriched ZnMMT system suggesting an influence of the type of organic molecule on the zinc release process.

### 2.5. Antimicrobial Activity

The inhibition zones produced by thymol, carvacrol, and their composite materials, evaluated at a concentration of 50 mg mL^−1^ of organic molecule, are shown in Table 3 and Table 4. One-way ANOVA analysis showed a significant effect (*p* ≤ 0.05) of the type of organic molecules or their composite materials on the inhibition zone values. Carvacrol and thymol produced inhibition zones against all spoiler and pathogenic strains, but with higher values against pathogenic bacteria than spoilage bacteria. The most sensitive strains were *Erwinia persicina* ITEM 17997 and *Staphylococcus aureus* DSM 799, whereas the most resistant strains were *Pseudomonas chicorii* ITEM 17296 and *P. aeruginosa* DSM 939, for spoiler and pathogenic strains, respectively. Clays (MMT or ZEO) did not show antibacterial activity against all target strains. MMT and ZEO–clay hybrids loaded with carvacrol and thymol showed, in most cases, inhibition zones comparable to pure compounds. MMT–carvacrol showed inhibition zones significantly higher than carvacrol for *P. chicorii* ITEM 17296 and *Escherichia coli* ATCC 35401, whereas MMT–thymol showed inhibition zones significantly higher than thymol for all spoiler strains, and also for pathogenic strains of *E. coli* ATCC 35401, *Listeria monocytogenes* DSM 20600, and *Sta. aureus* DSM 799 (Table 3). ZEO–carvacrol showed inhibition zones significantly higher than carvacrol for *P. putida* ITEM 17297, *P. chicorii* ITEM 17296, *P. aeruginosa* DSM 939, *Salmonella enterica* ATCC 13311, and *Sta. aureus* DSM 799, whereas ZEO–thymol showed inhibition zones significantly higher than thymol for *P. chicorii* ITEM 17296, *P. aeruginosa* DSM 939, and *Sta. aureus* DSM 799 (Table 4).

Clay hybrids loaded with essential oil compounds such as thymol and carvacrol showed improved antibacterial activity as compared to pure essential oil compounds. However, few studies evaluated the antibacterial activity of clay hybrids similar to those characterized in our work. In particular, Cometa et al. [12] found that ZEO4A/thymol hybrid showed better antibacterial activity than thymol, at the same concentration of the essential oil compound (75 mM), against *P. aeruginosa* DSM 939. Bernardos et al. [41] found lower MIC values of MMT loaded with thymol, eugenol, or carvacrol than pure compounds against *Sta. aureus*. MMT-Thy was in most cases more active than MMT-Car against bacterial strains. This result was also confirmed by Zhong et al. [42] with palygorskite loaded with thymol or carvacrol against *E. coli* and *Sta. aureus*.

Regarding Zn-modified clays, ZnZEO showed no inhibition zones against target strains, whereas ZnMMT showed inhibition zones against all strains except for *P. aeruginosa* DSM 939. ZnMMT–carvacrol produced inhibition zones significantly higher than MMT–carvacrol for all strains, except *Sal. enterica* ATCC 13311. ZnMMT–thymol produced inhibition zones significantly higher than MMT–thymol for many of strains, except for *Erw. persicina* ITEM 17997, *E. coli* ATCC 35401, *P. aeruginosa* DSM 939, and *Sta. aureus* DSM 799 (Table 3). Zn-modified ZEO hybrids showed in most cases a reduction in antibacterial activity compared to ZEO hybrids without zinc. An improvement in antibacterial activity was detected for ZnZEO–carvacrol against *E. coli* ATCC 35401 and *Sta. aureus* DSM 799, and for ZnZEO–thymol against *Erw. persicina* ITEM 17997, *P. putida* ITEM 17297, *E. coli* ATCC 35401, and *L. monocytogenes* DSM 20600. The inhibition zones produced by clay hybrids were generally improved in Zn-modified clay hybrids. In this respect, carvacrol incorporated onto ZnO/palygorskite nanoparticles showed lower MIC values against *E. coli* than carvacrol/palygorskite composite material [43]. The most interesting result we found was the different antibacterial activity of Zn-modified ZEO and MMT hybrids. ZnMMT hybrids always showed better antimicrobial performances than ZnZEO hybrids. This result could be explained by the higher zinc release from the MMT hybrids than ZEO clay materials. Regarding the antibacterial mechanism of action, Zn and monoterpenes could act synergically targeting the structural integrity of the cell, the electron transport system, various enzymatic activities, the nucleic acid, protein and cell wall synthesis, and membrane functionality [2,44].

Our findings are in accordance with those of Li et al., which demonstrated the improving in antibacterial activity of *Eucalyptus citriodora* essential oil, lacking in both carvacrol and thymol, in the presence of zinc ions against *E. coli* O157:H7 [45]. Conversely, Windiasti et al. demonstrated a synergistic effect of carvacrol and ZnO nanoparticles against *Campylobacter jejuni*, where carvacrol damaged the cell membrane followed by the cell leakage induced by ZnO [46].

To compare ZEO and MMT hybrids, the ratio among inhibition zones of hybrids against pure organic molecule was calculated (Table 5). Zn-modified MMT hybrids generally showed average antibacterial activity higher than that displayed by Zn-modified ZEO hybrids.

For this reason, a deep evaluation of the antibacterial activity of Zn-modified MMT clays was performed through the determination of MIC values in comparison to pure carvacrol and thymol, loading blank disks with of 20 μL of different samples in the range of 50–3.15 mg mL^−1^ of organic molecule.

The results of these assays are shown in Table S1 of the Supplementary Materials.

Carvacrol and thymol showed a MIC value lower than 3.15 mg mL^−1^ for *Erw. persicina* ITEM 17997 and *Pectobacterium carotovorum* subsp. *carotovorum* LMG 2404, and of 25 mg mL^−1^ against *P. putida* ITEM 17297 and *P. chicorii* ITEM 17296. For bacterial pathogens, carvacrol showed a MIC value lower than 3.15 mg mL^−1^ for both *E. coli* strains and *Sta. aureus* DSM 799, of 6.3 mg mL^−1^ against *L. monocytogenes* DSM 20600 and *Sal. enterica* ATCC 13311, and of 25 mg mL^−1^ against *P. aeruginosa* DSM 939; thymol showed a MIC value of 6.3 mg mL^−1^ against *P. aeruginosa* DSM 939, and lower than 3.15 mg mL^−1^ for the remaining pathogens. In the case of ZnMMT loaded with carvacrol and thymol, MIC values were lower than 3.15 mg mL^−1^ for all strains. Only *P. aeruginosa* DSM 939 showed higher MIC values, specifically 25 mg mL^−1^ for ZnMMT–carvacrol and 12.5 mg mL^−1^ for ZnMMT–thymol (Table S1).

The comparison of the antimicrobial activity between carvacrol and thymol showed that the average activity displayed by carvacrol for all concentrations and for all target strains resulted to be the 62.70 ± 17.76% for pathogens (range of 31.88–86.29%) and 84.85 ± 32.40% for spoilers (range of 59.62–132.42%).

**Figure 8:**
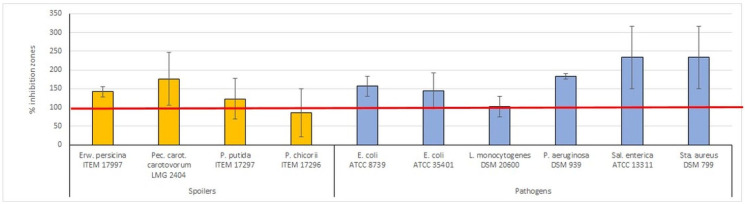
Ratio percentage between the average inhibition zone of ZnMMT–thymol for all concentrations and the average inhibition zone of ZnMMT–carvacrol for all target strains. The red line represents the 100% activity played by ZnMMT–carvacrol.

To understand the effect of the hybrid material type (ZnMMT–carvacrol or ZnMMT–thymol) on the inhibition zones values, measured at 25 mg mL^−1^ of carvacrol or thymol equivalent concentration, the Shapiro–Wilk test followed by a bilateral T Student test was applied. For both spoiler and pathogenic strains, replicate values of inhibition zones, produced by ZnMMT–carvacrol and ZnMMT–thymol, were significantly different for *p* < 0.05. On these premises, the average inhibition zones of ZnMMT–carvacrol and ZnMMT–thymol were compared independently by the carvacrol- or thymol-equivalent concentration showing higher antibacterial activity of ZnMMT–thymol than ZnMMT–carvacrol against eight out of ten bacterial strains (Figure 8).

**Figure 9:**
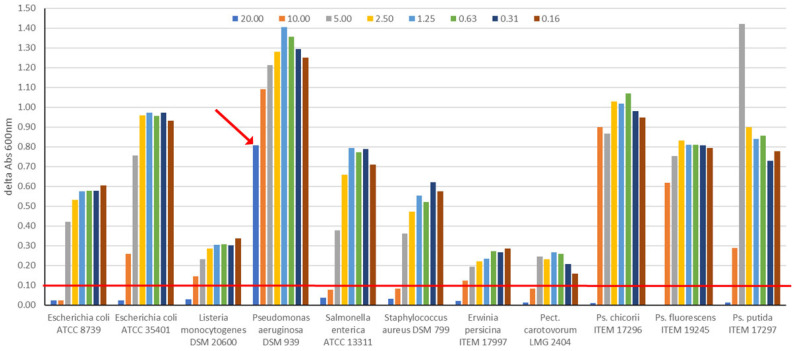
Difference in absorbance values between the end and the beginning of incubation of pathogenic and spoilage bacteria grown in broth culture in the concentration range of 50.0–0.0 mg/mL of thymol.

Based on these results, antimicrobial activity assays moved from the measurement of inhibition zones to the calculation of minimal inhibitory concentration (MIC) of pure thymol and ZnMMT–thymol in broth culture medium. The MIC values were obtained by means of the difference in absorbance values (OD_600nm_) between the beginning and the end of incubation, attributing the presence of growth when this difference was ≥0.1 (Figure 9).

Thymol produced MIC values of 20 mM (corresponding to 3 mg thymol mL^−1^) against *E. coli* ATCC 35401, *L. monocytogenes* DSM 20600, *Erw. persicina* ITEM 17997, *P. chicorii* ITEM 17296, *P. fluorescens* ITEM 19245, and *P. putida* ITEM 17297, and of 10 mM (corresponding to 1.5 mg thymol mL^−1^) against *E. coli* ATCC 8739, *Sal. enterica* ATCC 13311, *Sta. aureus* DSM 799, and *Pec. carotovorum* sub. *carotovorum* LMG 2404. The MIC value for *P. aeruginosa* DSM 939 was higher than 20 mM thymol, as highlighted by the red arrow in Figure 9. The viability assay carried out at the end of 24 h incubation informed us that thymol produced MBC values of 20 mM against all strains, except for *Erw. persicina* ITEM 17997 and *P. aeruginosa* DSM 939, which were more resistant (MBC values higher than 20 mM).

Based on these values, a new test was set up aiming at the evaluation of Zn–MMT–thymol antibacterial activity. To compare the thymol antibacterial performance with that of ZnMMT–thymol, the average loading of thymol in Zn–MMT was considered 28.5%. The tests were therefore carried out at the same thymol concentration in hybrid material as those of pure thymol. Due to the turbidity produced by ZnMMT–thymol, we proceeded to measure the viable cells directly in the control broth medium and in that supplemented with ZnMMT–thymol at 0.5xMIC values. The results are reported in Table 6.

The ZnMMT–thymol showed stronger antimicrobial activity than thymol in solution, probably due to the presence of the Zn ions loaded in MMT that were largely released during 24 h at 37 °C (Figure 6 and Figure 7). At the thymol concentration of 20 mM (3 mg mL^−1^), *P. aeruginosa* were still alive at a load of approximately 8 log cfu mL^−1^, while at the same concentration of thymol included in the ZnMMT–thymol, no viable cells were found. We can guess that this improvement is the result of synergistic or additive antimicrobial activity between thymol and zinc ions.

## 3. Materials and Methods

### 3.1. Materials

Zn(NO_3_)_2_ × 6H_2_O, Zn(CH_3_COO)_2_, thymol (2-isopropyl-5-methylphenol) (purity ≥98.5%), carvacrol (5-isopropyl-2-methylphenol) (purity 98%), and montmorillonite K10 (coded as MMT) were purchased from Sigma Aldrich (Milan, Italy). Zeolite 4A (coded as ZEO4A, purity >98%, D_50_ 3.5 µm), having an interconnected 3D network of channels approximately 4 Å in diameter, in addition to larger “cages” approximately 7 Å in diameter, was supplied by Nachmann S.r.l. (Milan, Italy).

### 3.2. Development of Zn-Modified Clays

Zn-modified montmorillonite (ZnMMT) was obtained via an ion exchange reaction starting from montmorillonite MMT K10. On a laboratory scale, the ion exchange reaction was carried out following the protocol reported by Dakovic et al. [47], with slight modifications. The reaction occurred starting from 1 g of MMT-K10 in 50 mL of a solution 0.14 M of zinc nitrate hexahydrate [Zn(NO_3_)_2_ × 6H_2_O] placed in a flask under magnetic stirring for 3 h at 60 °C. Subsequently, the solid was centrifuged to separate the ZnMMT solid from the supernatant, washed with distilled water, centrifuged, and placed in an oven at 80 °C until constant weight was reached.

To prepare the Zn-modified zeolite (ZnZEO) via formation of ZEO-ZnO nanocomposite, referring to Nyankson et al. [48], 4 g of zinc acetate was dissolved in 50 mL of distilled water, to which 2 g of ZEO4A was added, leaving it under stirring for 20 h. At the end of the reaction, 50 mL of a 0.1 M NaOH solution was added for 30 min to precipitate the Zn^2+^ ions. The precipitate was filtered and washed with distilled water and subsequently dried at 80 °C overnight. Then the precipitate was subjected to calcination at 500 °C for 2 h in a muffle.

### 3.3. Loading of the Zn-Modified Clays with Thymol or Carvacrol

In this work, the loading of thymol or carvacrol in Zn-modified clays was performed according to a solvent-free procedure reported in our previous work [12]. Moreover, composites (clay-bioactive molecule) without zinc were developed with the same procedure to ascertain the role of zinc on the antimicrobial properties.

Briefly, for thymol loading, since the active molecule was solid and possesses a low melting temperature, a melting/crystallization cycle in the presence of the clay was performed. In particular, the procedure consisted of a thermal treatment of thymol/zeolite physical mixture (ratio clay/active principle equal to 1:0.5 *w*/*w*) at 55 ± 2 °C for 10 min in an oven and subsequent rapid solidification at room temperature. In the case of carvacrol, which is liquid at room temperature, a physi-adsorption method was carried out, a mechanical incorporation of the oily liquid into the clay powder, using a mortar and pestle at room temperature. In this case, still needing to have a powder rather than a paste, composites with a clay/active molecule ratio equal to 1:0.25 *w*/*w* were developed.

### 3.4. Chemical–Physical Characterization of the Zn-Modified Clays

The developed clays were deeply characterized by FT-IR (ATR mode), TGA, XRD, and XPS analyses.

Fourier transform infrared spectroscopy in attenuated total reflectance mode, FT-IR (ATR), was carried out by means of a Spectrum Two PE instrument (PerkinElmer Inc., Waltham, MA, USA) endowing the universal ATR accessory (UATR, Single Reflection Diamond/ZnSe). FT-IR/ATR spectra were recorded in the range between 400 and 4000 cm^−1^ (resolution 4 cm^−1^).

Thermogravimetric analysis (TGA) was performed on a PerkinElmer TGA-400 instrument (PerkinElmer Inc., Waltham, MA, USA), heating 5–10 mg of samples in air-saturated atmosphere, and setting the gas flow to 20 mL/min. The flow rate was set to 20 °C/min, heating from 30 to 800 °C. The sample mass was in the range 5–15 mg. For each sample, thermograms (TGs) with respective derivative (DTG) curves were recorded and analyzed with TGA Pyris software.

X-ray powder diffraction (XRD) profiles were acquired with a Miniflex II (Rigaku Corporation, Tokyo, Japan) X-ray diffractometer equipped with a Cu Kα X-ray source (30 kV, 15 mA) and a secondary beam graphite monochromator. All the measurements were recorded from 2θ = 5° to 65° at a scan speed of 0.01°/s and continuous spin of the sample. XRD data analysis and ICDD (International Centre for Diffraction Data) database matching were performed with the software PDXL v. 1.8.1.0 (Rigaku Corporation, Tokyo, Japan) using PDF-2 ICDD database.

XPS analyses were performed using a scanning microprobe PHI 5000 VersaProbe II purchased from Physical Electronics (Chanhassen, MN, USA). The instrument was equipped with a micro-focused monochromatized AlKα X-ray radiation source. Samples were examined in HP mode with an X-ray take-off angle of 45°, with an instrument base pressure ~10^−9^ mbar. The scanned area sizes were 1400 × 200 μm. Wide scans and high-resolution spectra were recorded in FAT mode (pass-energy equal to 117.4 eV and 29.35 eV, respectively). For curve-fitting of the high-resolution spectra, the commercial MultiPak software version 9.9.0.8 was used. Adventitious carbon C1s was set as the reference charge (284.8 eV).

### 3.5. Quantification of Thymol and Carvacrol by GC-MS

GC-MS analyses were carried out to determine thymol or carvacrol content within the composites. For the preparation of the samples, the composites were dispersed in ethanol at a concentration of 1 mg/mL (mass of the composite/volume of ethanol). In the ethanol extracting medium, the internal standard was also added, at a concentration of 250 μg/mL. Thymol was used as an internal standard for carvacrol and, symmetrically, carvacrol was used as an internal standard for thymol. The calibration curves obtained demonstrated an R^2^ value always higher than 0.997 units. Each calibration level was fortified with the internal standard at a constant concentration of 2.5 μg/mL. For each sample, three extraction replicates were produced. The extraction was favored by vigorous vortexing, followed by sonication for 5 min. Subsequently, the mixture was subjected to centrifugation for 10 min at 4500 rpm. The supernatant, containing the extracted thymol or carvacrol, was then diluted by a factor of 1:100, followed by characterization by GC-MS.

A gas chromatographic method was developed to guarantee a rapid and efficient separation of the two isomers. The gas chromatographic separation involved the use of a 30 m Elite 5-MS column (Perkin Elmer), with an internal diameter of 250 μm and a stationary phase thickness of 0.25 μm. The separation was managed by the Clarus 680 gas chromatograph (Perkin Elmer), operating in isothermal mode for a total duration of 8 min at a temperature of 135 °C. Helium was used as the mobile phase at a flow of 1 mL/min, adopting a split ratio of 1:10. The sample injection took place manually, with reference to a sample volume equal to 1 μL. The injection chamber was maintained at a temperature of 350 °C for the entire duration of the gas chromatographic separation. The detection of the target analytes occurred by mass spectrometry, using a single quadrupole Clarus SQ 8 T (Perkin Elmer) spectrometer. Specifically, single ion monitoring (SIM) mode was used to maximize the sensitivity of the analytical method. For both carvacrol and thymol, the *m*/*z* 135 ion, resulting from the neutral loss of a methyl radical from the molecular ion ([M − CH_3_]^+^), was used as a quantifier ion. For both isomers, in fact, this signal corresponded to the base peak within the relevant EI-MS spectra. The molecular ion (*m*/*z* 150), however, was used as a qualifier ion. The operation of the quadrupole provided dwell time and inter-channel delay values equal to 0.1 s and 0.02 s, respectively. Furthermore, a solvent cut time of 5 min was set. The ionization of the eluting species occurred by electron ionization (EI). The source operated in positive mode.

For each composite, the loading content and loading efficiency percentages (LC% and LE%, respectively) were calculated as follows:

LC% = (m_Loaded AM_/m_composite_) × 100

LE%= (m_Loaded AM_/m_Expected AM_) × 100

where m_Loaded AM_ and m_Expected AM_ are the masses of the active molecule (thymol or carvacrol) measured after the composite preparation and of thymol or carvacrol added during the preparation, respectively, while the m_composite_ was the mass of the analyzed composite.

In addition, in vitro release tests were performed, in triplicate, immersing 5 mg of the composites in 5 mL of distilled water up to 7 days at 37°. Then, thymol or carvacrol released amounts were detected by GC-MS at 24 h and 7 days after an extraction with hexane.

### 3.6. Quantification of Zinc by Spectrophotometric Analysis

The evaluation of zinc loading and its release from ZnMMT and ZnZEO systems were carried out in aqueous medium using the spectrophotometric procedure reported by Wang et al. [49]. Zinc release from Zn-modified clays loaded with thymol or carvacrol was also investigated to ascertain if the presence of the organic molecules influences the metal ions release. In each experiment, 5 mg of composite were dipped in 5 mL of distilled water at T room. Successively, for the colorimetric reaction, aliquots of 500 µL were withdrawn and replaced with an equal amount of water after 24 h and 7 days of immersion. The zinc concentration after 7 days was corrected using the following formula considering the dilution effect introduced after the withdrawal at 24 h:

$$C_{{\left(7d\right)}_{corr}}=C_{7d}+C_{24h}\frac{V_{W}}{V_{0}}$$

where *C*_24h_ and *C*_7d_ are the zinc concentration measured after 24 h and 7 days, *V_W_* is the withdrawal volume (500 µL), and *V*_0_ is the total volume (5 mL).

Each experiment was carried out in triplicate and data were reported as mean value ± SD.

### 3.7. Antioxidant Activity Evaluation of Thymol and Carvacrol

Thymol and carvacrol in vitro antioxidant activity was tested via DPPH and ABTS assays, according to the protocols described by Luo et al. [50]. Briefly, DPPH solution (100 µM) was prepared in methanol and its absorbance was measured at 517 nm. Calibration curves (r2 = 0.999) were obtained with thymol or carvacrol standard solutions (2 to 25 ppm). An amount of 3 mL of standard or sample solution was mixed with 1 mL of DPPH solution and their absorbance was measured at 517 nm. The radical scavenging activity percentages (% RSAs) were calculated with the following equation:

% RSA = (Arad − A_S_)/Arad × 100

in which A_S_ represents the sample’s absorbance, whereas Arad is the absorbance of the bare DPPH radical. Each measurement was performed in triplicate and expressed as mean ± standard deviation. Ascorbic acid (50 µM) scavenging activity on DPPH radicals was also assessed and compared to the extract’s activity. All the assays were performed using a UV–visible Spectrophotometer UV-1900i, (Shimadzu, Milan, Italy).

Regarding the ABTS test, the radical ABTS was dissolved in PBS (0.01 M, pH 7.4) at a concentration of 7 mM, the radical cation was obtained after 16 h of reaction in the dark with 2.45 mM ammonium persulfate (APS), and it was then diluted to absorbance 0.70 ± 0.02 at 734 nm before use. An amount of 0.2 mL of the sample dissolved in ethanol (in the range 1–300 μg/mL), mixed with 2.0 mL of ABTS+, and the absorbance was measured at 734 nm after 6 min. The antioxidant activity was calculated using the following equation:

ABTS scavenging effect (%) = [A0 − (As − Ab)]/A0 × 100

where A0 is the ABTS+ absorbance, As is the absorbance of ABTS+ with the sample, and Ab is the absorbance of the sample without the radical cation.

Furthermore, the DPPH and ABTS tests were also performed on Zn-modified clays after the loading of thymol or carvacrol. Composite samples were extracted in methanol or ethanol for DPPH or ABTS assays, respectively. Different extractions times were evaluated from 0 to 24 h; however, we observed no changes in the extraction performances depending on the time since methanol and ethanol proved to be good solvents for solubilizing both analytes and a complete extraction was obtained immediately. However, to allow for the correct comparison between free thymol or free carvacrol activity and that of the same molecules loaded in Zn-modified clays, the tests were performed considering the loading percentages of thymol or carvacrol in the clays evaluated by GC-MS (Section 3.5).

### 3.8. Bacterial Strains and Growth Conditions

The antibacterial activity of all molecules and hybrids was assessed against 4 spoiler and 5 pathogenic bacteria. Bacteria were previously isolated from plant foods or were purchased from international bacterial collections including BCCM/LMG Bacteria Collection, Leibniz-Institut DSMZ, German Collection of Microorganisms and Cell Cultures, and Agro-Food Microbial Culture Collection (ITEM) at the Institute of Sciences of Food Production of Bari, Italy (http://server.ispa.cnr.it/ITEM/Collection/, accessed on 30 June 2024).

Spoilage bacteria included *Erwinia persicina* ITEM 17997 [51], *Pectobacterium carotovorum* subsp. *carotovorum* LMG 2404, *Pseudomonas putida* ITEM 17297, and *Pse. chicorii* ITEM 17298 [52], whereas pathogenic bacteria included *Escherichia coli* ATCC 8739, *E. coli* ATCC 35401, *Listeria monocytogenes* DSM 20600, *Pse. aeruginosa* DSM 939, *Salmonella enterica* ATCC 13311, and *Staphylococcus aureus* DSM 799.

Spoiler strains were grown in mPlate Count Broth (mPCB, Becton Dickinson Italia, Milan, Italy) overnight at 30℃, whereas pathogens were grown in Brain Heart Infusion broth (BHI, Biolife Italiana Srl, Milan, Italy) overnight at 37 °C.

### 3.9. Evaluation of Antibacterial Activity

Thymol, carvacrol, as well as all loaded and/or Zn-modified clay hybrids, were assayed for their antimicrobial activity by using a disk diffusion test [53] against target strains reported in Section 3.8.

After overnight incubation, fresh microbial suspensions were centrifuged (5000 × *g* for 5 min), diluted in sterile saline solution (9 g L^−1^ NaCl) to OD_600_ value of 0.3 ± 0.05 (ca. 8 log CFU mL^−1^), and further diluted in sterile saline solution to approximately 6 log CFU mL^−1^. A volume of 100 microliters of this suspension was spread with a sterile loop over agar plates containing plate count agar (PCA).

To compare antimicrobial activities of loaded/Zn-modified clays and pure thymol, carvacrol, clay hybrids were suspended in n-hexane at the same estimated final concentration of organic compound. Following this approach to obtain 50 mg/mL of thymol or carvacrol, loaded hybrids were suspended in n-hexane in the range of 37,9–82,6 mg/mL depending on different hybrid loading capacities.

A sterile paper disc (diameter 6 mm, Biolife Italiana S.r.l., Milan, Italy) was laid over the solidified substrate and charged with 20 μL of each sample diluted in n-hexane at different concentrations. Disks amended with 20 µL of n-hexane were used as negative controls. Plates with target strains and disks were firstly refrigerated at 4 °C for 2 h and then incubated kept at 30 °C or 37 °C for 24 h. After the incubation, the areas of the inhibition zones (expressed in mm^2^), net of those of the disks, were calculated using image acquisition software and standardized on the basis of the area of six surface markers in the range of 28.2 to 530.9 mm^2^, present in each picture, as previously reported [54].

The antimicrobial activity of ZnMMT–carvacrol and ZnMMT–thymol hybrids was further evaluated in the range of thymol- or carvacrol-equivalent concentration of 50,0–3125 mg/mL. Under these conditions, the minimum inhibitory concentration (MIC) was defined as the lowest concentration producing a measurable inhibition zone.

All assays were performed in three replicates.

Minimum inhibitory concentration (MIC) and minimum bactericidal concentration (MBC) values of thymol were defined after incubation of target strains in broth supplemented with different thymol concentrations. A stock solution of thymol (100 mM) in 5% ethanol was prepared. Then, dilutions in plate count broth (PCB) were made to reach the final concentrations of 20, 10, 5, 2.5, 1.25, 0.625, and 0.3125 mM, corresponding to 3.00, 1.50, 0.75, 0.38, 0.19, 0.09, and 0.05 mg mL^−1^.

Microbial broth was inoculated with a fresh viable cell culture to initial cell density of approximately 4 log CFU mL^−1^. For each strain, 1.5 mL of this suspension was distributed into a 48-well multiplate; broth without the addition of thymol was used as positive control. Plates were incubated for 24 h at 30 °C or 37 °C for spoilage bacteria and pathogens, respectively, reading the absorbance 600 nm value through the Varioskan Flash instrument (ThermoFischer Scientific, Waltham, MA, USA) at the beginning and after 24 h of incubation. The MIC value was defined as the lowest concentration showing a difference in absorbance values (t24–t0) less than 0.1. The MBC values were determined through a viability assay. A volume of 50 μL of samples without visible bacterial growth were inoculated into 1 mL of PCB, and incubated for 24 h at 30 °C or 37 °C. The absence of microbial growth informed about bactericidal concentration.

After the determination of MIC and MBC values of thymol, the rate of cell death was defined in broth with the addition of ZnMMT–thymol hybrid at 0.5xMIC values thymol. After incubation for 24 h at 30 °C or 37 °C, cell suspensions were serially diluted in sterile saline solution (9 g L^−1^ NaCl) and plated on PCA plates. Plates were incubated at optimum temperature for 24 h measuring viable cell loads that were expressed as log cfu mL^−1^.

### 3.10. Statistical Data Analysis

One-way Student’s *t*-test analysis was applied to estimate differences between each organic molecule or composite materials in terms of antioxidant activity (*p* ≤ 0.05). One-way ANOVA was applied to estimate the effect of the type of organic molecule or composite material on inhibition zone (*p* ≤ 0.05). Duncan post-hoc test was applied to differentiate mean values.

## 4. Conclusions

In this study, montmorillonite and zeolite modified with zinc ions were loaded with carvacrol or thymol to obtain antibacterial hybrids. The idea was to potentiate the antimicrobial activity of the bioactive molecules by the presence of zinc species, well-known for their antimicrobial properties, to obtain highly active formulations against various microbial target strains.

Physicochemical characterization of the hybrids demonstrated that, despite to the lower zinc amount recorded by XPS, the ZnMMT composite showed the higher zinc release, in agreement with its antimicrobial behavior. An influence of the type of organic molecule on the zinc release process was also observed. After 24 h, ZnMMT–thymol released large part of thymol in distilled water, whereas after 7 days, all composites released approximately the total loaded amount of organic molecules.

When hybrid materials were evaluated for their antimicrobial activity, several differences were found. The best results were produced by the MMT hybrid loaded with Zn and functionalized with thymol. In vitro assays demonstrated that antimicrobial activity of ZnMMT–thymol employed at the same thymol concentration was widely stronger than that of pure thymol. Based on zinc release data from the same composite this improvement was the result of a synergistic or additive antimicrobial activity between thymol and zinc ions.

The new ZnMMT–thymol here defined can be used for several downstream applications for new products for microbial control in several sectors.

## Figures and Tables

**Table 1:** Atomic percentages detected by XPS analysis on the surface of the clays.

Clay	Atomic Percentage (%)								
	C1s	O1s	Al2p	Si2p	Mg1s	Ca2p	Na1s	N1s	Zn2p_3/2_
MMT	3.9	67.3	4.0	22.8	1.5	0.5	--	--	--
ZnMMT	4.1	67.4	4.1	22.7	1.1	--	--	--	0.6
ZEO	21.9	45.7	9.9	9.2	--	--	13.3	--	--
ZnZEO	15.4	54.4	8.4	9.3	--	--	--	--	12.4

**Table 2:** Carvacrol and thymol concentrations in the different composites measured by GC-MS. Loading capacity (LC%) and loading efficiency (LE%) percentages were also reported.

Hybrid Material	μg/mL	LC%	LE%
MMT–carvacrol	272 ± 12	27.2%	136%
ZEO–carvacrol	258 ± 1	25.8%	129%
ZnMMT–carvacrol	249 ± 23	24.9%	125%
ZnZEO–carvacrol	246 ± 11	24.6%	123%
MMT–thymol	331 ± 10	33.1%	99%
ZEO–thymol	306 ± 7	30.6%	92%
ZnMMT–thymol	347 ± 12	34.7%	104%
ZnZEO–thymol	363 ± 21	36.3%	109%

**Table 3:** Inhibition zones (mm^2^) produced by MMT ion exchanged with Zn or loaded with organic molecules or both against spoiler and pathogenic strains. Different lowercase letters indicate significant differences (*p* ≤ 0.05) among each strain.

		Carvacrol	Thymol	MMT–Carvacrol	MMT–Thymol	ZnMMT	ZnMMT–Carvacrol	ZnMMT–Thymol
*Spoilers*	*Erw. persicina* ITEM 17997	117.40 c ± 10.60	165.10 b ± 14.80	93.60 c ± 8.50	220.70 a ± 12.80	46.42 d ± 16.92	234.60 a ± 20.60	218.70 a ± 8.30
	*Pec. carot. carot.* LMG 2404	369.30 b ± 15.70	256.60 d ± 16.60	122.00 e ± 25.00	379.30 b ± 38.30	69.67 f ± 15.67	304.74 c ± 13.74	648.44 a ± 22.56
	*P. putida* ITEM 17297	91.20 c ± 20.80	82.02 c ± 8.98	48.50 d ± 11.50	128.60 b ± 10.60	64.91 cd ± 3.91	249.80 a ± 24.20	275.80 a ± 15.80
	*P. chicorii* ITEM 17296	47.20 f ± 8.20	60.20 e ± 5.50	93.60 d ± 4.50	120.70 c ± 3.20	41.29 ef ± 8.21	208.70 b ± 14.20	504.30 a ± 12.50
								
*Pathogens*	*E. coli* ATCC 8739	543.28 b ± 11.02	432.43 c ± 39.07	315.13 d ± 14.03	417.96 c ± 17.16	61.89 e ± 5.01	416.44 c ± 13.74	631.31 a ± 29.81
	*E. coli* ATCC 35401	238.7 d ± 20.8	205.34 e ± 13.84	412.75 c ± 13.45	541.84 a ± 13.43	92.89 f ± 2.79	491.67 b ± 6.77	391.68 c ± 10.48
	*L. monocytogenes* DSM 20600	267.74 c ± 38.44	133.77 d ± 18.87	299.87 c ± 14.43	161.87 b ± 10.57	296.17 c ± 18.73	468.68 d ± 18.58	825.16 a ± 63.84
	*P. aeruginosa* DSM 939	10.08 c ± 1.92	58.1 b ± 3.4	7.05 c ± 3.05	51.31 b ± 3.31	0 c ± 0	73.75 a ± 10.85	61.26 ab ± 18.54
	*Sal. enterica* ATCC 13311	554.76 a ± 13.26	374.8 d ± 13.6	471.54 b ± 26.74	393.54 d ± 6.44	91.43 e ± 9.57	430.59 c ± 10.41	541.08 a ± 13.12
	*Sta. aureus* DSM 799	3877.67 a ± 76.83	2418.31 d ± 197.09	3308.81 c ± 117.71	3547.65 b ± 160.55	53.62 e ± 15.38	4022.11 a ± 18.89	2240.16 d ± 114.04

One-way ANOVA analysis was applied to estimate the effect of the type of organic molecule or composite material on inhibition zone (*p* ≤ 0.05). Duncan post-hoc test was applied to differentiate mean values. Different lowercase letters indicate significant differences (*p* ≤ 0.05) among each strain.

**Table 4:** Inhibition zones (mm^2^) produced by ZEO ion exchanged with Zn or loaded with organic molecules or both against spoiler and pathogenic strains. Different lowercase letters indicate significant differences (*p* ≤ 0.05) among each strain.

		Carvacrol	Thymol	ZEO–Carvacrol	ZEO–Thymol	ZnZEO	ZnZEO–Carvacrol	ZnZEO–Thymol
*Spoilers*	*Erw. persicina* ITEM 17997	117.40 b ± 10.60	165.10 a ± 14.80	82.60 c ± 4.60	101.00 bc ± 40.50	0.00 d ± 0.00	79.60 c ± 8.60	154.00 a ± 13.50
	*Pec. carot. carot.* LMG 2404	369.30 a ± 15.70	256.60 bc ± 16.60	284.70 b ± 22.30	270.20 b ± 29.00	0.00 d ± 0.00	218.50 c ± 11.00	245.71 bc ± 38.31
	*P. putida* ITEM 17297	91.20 b ± 20.80	82.02 bc ± 8.98	134.50 a ± 17.20	61.80 c ± 9.80	0.00 d ± 0.00	80.30 bc ± 7.80	93.30 b ± 6.00
	*P. chicorii* ITEM 17296	47.20 c ± 8.20	60.20 bc ± 5.50	82.60 ab ± 11.50	101.00 a ± 36.50	0.00 d ± 0.00	45.30 c ± 7.60	57.00 bc ± 7.20
								
*Pathogens*	*E. coli* ATCC 8739	543.28 a ± 11.02	432.43 b ± 39.07	478.10 b ± 31.10	281.13 d ± 51.23	0.00 e ± 0.00	364.39 c ± 10.51	292.05 d ± 11.85
	*E. coli* ATCC 35401	238.7 a ± 20.8	205.34 b ± 13.84	116.96 d ± 7.96	162.22 c ± 10.32	0.00 e ± 0.00	169.91 c ± 18.57	232.81 a ± 10.11
	*L. monocytogenes* DSM 20600	267.74 b ± 38.44	133.77 d ± 18.87	238.29 b ± 16.39	62.87 e ± 11.37	0.00 f ± 0.00	191.63 c ± 17.87	389.48 a ± 10.28
	*P. aeruginosa* DSM 939	10.08 d ± 1.92	58.1 b ± 3.4	46.78 c ± 9.42	121.02 a ± 9.82	0.00 d ± 0.00	5.02 d ± 0.98	49.60 bc ± 5.20
	*Sal. enterica* ATCC 13311	554.76 b ± 13.26	374.8 c ± 13.6	604.69 a ± 40.11	392.41 c ± 35.31	0.00 f ± 0.00	203.30 e ± 2.30	302.90 d ± 31.30
	*Sta. aureus* DSM 799	3877.67 c ± 76.83	2418.31 d ± 197.09	2427.07 d ± 135.97	5236.85 a ± 79.75	0.00 e ± 0.00	4024.46 c ± 266.54	4681.65 b ± 227.45

One-way ANOVA was applied to estimate the effect of the type of organic molecule or composite material on inhibition zone (*p* ≤ 0.05). Duncan post-hoc test was applied to differentiate mean values. Different lowercase letters indicate significant differences (*p* ≤ 0.05) among each strain.

**Table 5:** Ratio percentage between the average inhibition zones (mm^2^) of MMT and ZEO ion-exchanged with Zn and loaded with organic molecules against inhibition zones (mm^2^) produced by carvacrol or thymol against spoiler and pathogenic strains.

	Spoilers	Pathogens	Average
Zn–MMT–carvacrol/carvacrol	254.83 ± 8.59	234.34 ± 37.75	244.58 ± 14.49
Zn–MMT–thymol/thymol	392.20 ± 2.84	218.56 ± 28.84	305.38 ± 122.78
Zn–ZEO–carvacrol/carvacrol	79.17 ± 5.96	66.92 ± 3.64	73.04 ± 8.66
Zn–ZEO–thymol/thymol	99.84 ± 12.20	139.58 ± 3.75	119.71 ± 28.11

**Table 6:** Microbial counts (in log cfu mL^−1^) of spoilage and pathogenic bacteria after 24 h of incubation in control broth medium and in the presence of ZnMMT–thymol added at 0.5xMIC thymol concentration.

		Control		0.5xMIC	% Mortality
**Spoilres**	*Erw. persicina* ITEM 17997	8.18 ± 0.12		nd	100.00
	*Pec. carotovorum* LMG 2404	7.33 ± 0.11		nd	100.00
	*P. chicorii* ITEM 17296	7.81 ± 0.08		nd	100.00
	*P. fluorescens* ITEM 19245	9.00 ± 0.36		nd	100.00
	*P. putida* ITEM 17297	8.48 ± 0.61		nd	100.00
					
**Pathogens**	*E. coli* ATCC 8739	8.00 ± 0.37		1.97 ± 0.04	99.99
	*E. coli* ATCC 35401	9.54 ± 0.56		3.96 ± 0.14	99.98
	*L. monocytogenes* DSM 20600	8.39 ± 0.11		nd	100.00
	*P. aeruginosa* DSM 939	10.10 ± 0.09		nd	100.00
	*Sal. enterica* ATCC 13311	9.29 ± 0.38		nd	100.00
	*Sta. aureus* DSM 799	8.70 ± 0.15		nd	100.00

nd: not detected; level of detection 1 log cfu mL^−1^.

## Data Availability

The data presented in this study are available from the corresponding authors on reasonable request.

## Supplementary Materials

The following supporting information can be downloaded at: https://www.mdpi.com/article/10.3390/molecules29153607/s1, Figure S1: Total ion current chromatogram (TICC) for the separation of an equimolar mixture of carvacrol and thymol. The TICC chromatogram corresponds to the sum of the contributions of the GC-MS traces recorded in SIM mode for the *m*/*z* 150 and *m*/*z* 135 ions; Figure S2: The EI-MS spectra of thymol (A) and carvacrol (B) averaged below the corresponding chromatographic band in the gas chromatographic trace acquired in FullMS scan mode. In both images, the *m*/*z* ratios of the ions used as quantifier and qualifier for both thymol and carvacrol were highlighted (see Section 2.5); Table S1: Inhibition zones produced by Zn-MMT hybrids or pure carvacrol and thymol, at different concentration (20 µL of 50–3.15 mg mL^−1^ carvacrol or thymol equivalent concentration) against spoiler and pathogenic strains.
