# Supplementary material for: Analytical and Antimicrobial Characterization of Zn-Modified Clays Embedding Thymol or Carvacrol
Source: Molecules. 2024 Jul 30;29(15):3607. doi: 10.3390/molecules29153607 (PMC11313700; doi:10.3390/molecules29153607)
Supplement: Supplementary file 1 [file molecules-29-03607-s001.zip › molecules-3109598-supplementary.pdf]

Supporting material

Analytical and antimicrobial characterization of Zn-modified clays embedding thymol or carvacrol

Loris Pinto[1#], Federico Baruzzi[1#], Roberto Terzano[2], Francesco Busto[3,4], Alessia Marzullo[1], Carmela Magno[5], Stefania Cometa[6*], Elvira De Giglio[3,4*]

[1] Institute of Sciences of Food Production, CNR, Via G. Amendola 122/O, 70126, Bari, Italy
[2] Department of Soil, Plant and Food Sciences, University of Bari, Via Orabona 4, 70126, Bari, Italy
[3] Department of Chemistry, University of Bari, Via Orabona 4, 70126, Bari, Italy
[4] Consorzio INSTM, Via Giusti 9, 50121 Firenze, Italy
[5] VIBAC SpA, Strada Ticineto Salita San Salvatore 40, 15040, Ticineto (AL), Italy
[6] Jaber Innovation s.r.l., Via Calcutta 8, 00144, Rome, Italy

* Correspondence: stefania.cometa@jaber.it and elvira.degiglio@uniba.it

[#] These authors equally contributed to the work

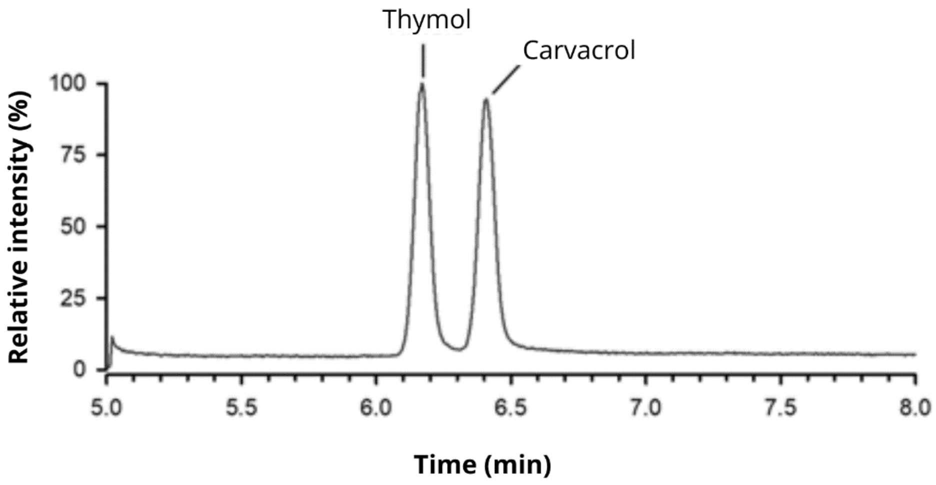

Figure S1: Total ion current chromatogram (TICC) for the separation of an equimolar mixture of carvacrol and thymol. The TICC chromatogram corresponds to the sum of the contributions of the GC-MS traces recorded in SIM mode for the m/z 150 and m/z 135 ions.

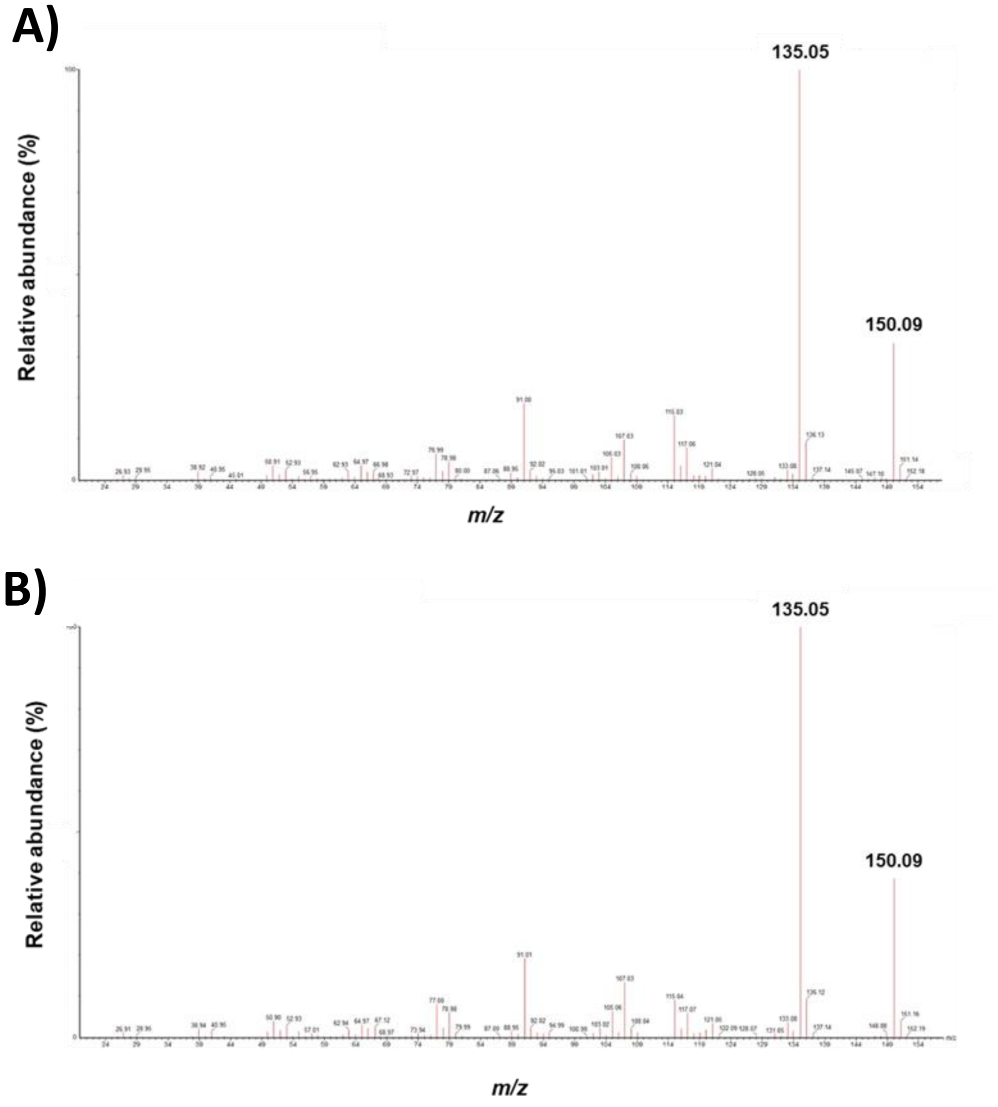

Figure S2: The EI-MS spectra of thymol (A) and carvacrol (B) averaged below the corresponding chromatographic band in the gas chromatographic trace acquired in FullMS scan mode. In both images, the m/z ratios of the ions used as quantifier and qualifier for both thymol and carvacrol were highlighted (see Section 2.6).

Table S1 Inhibition zones produced by Zn-MMT hybrids or pure carvacrol and thymol, at different concentration (20 µL of 50-3.15 mg mL-1 carvacrol or thymol equivalent concentration) against spoiler and pathogenic strains.

		Carvacrol					Thymol					**ZnMMT-carvacrol**					**ZnMMT–thymol**				
		50	25	12.5	6.3	3,15	50	25	12,5	6,3	3,15	50	25	12,5	6,3	3,15	50	25	12,5	6,3	3,15
Spoilers	*Erw. persicina* ITEM 17997	164.36 ± 4.96	58.12 ± 8.82	35.74 ± 2.74	28.64 ± 4.36	20.11 ± 2.11	296.62 ± 12.95	72.49 ± 4.51	62.41 ± 2.01	56.71 ± 6.41	36.75 ± 3.75	213.7 ± 25.8	176.91 ± 6.81	59.53 ± 14.93	38.23 ± 3.13	32.41 ± 1.69	249.9 ± 17.1	261.31 ± 14.31	110.61 ± 14.39	58.53 ± 3.13	44.11 ± 3.81
	Pec. carot. carot. LMG 2404	237.75 ± 11.25	179.91 ± 20.91	72 ± 11	48.24 ± 4.76	20.9 ± 1.1	291.62 ± 9.58	183.45 ± 8.05	104.92 ± 5.08	66.87 ± 12.57	42.76 ± 1.24	250.71 ± 16.69	97.54 ± 3.66	85.41 ± 2.69	90.52 ± 1.92	43.57 ± 6.57	224.3 ± 9.2	218.72 ± 7.38	204.21 ± 5.11	97.95 ± 1.05	95.9 ± 2.9
	P. putida ITEM 17297	96.4 ± 2.6	19.97 ± 1.03	0.00 ± 0.00	0.00 ± 0.00	0.00 ± 0.00	88.58 ± 10.42	12.8 ± 11.2	0.00 ± 0.00	0.00 ± 0.00	0.00 ± 0.00	127.3 ± 6.2	65.91 ± 8.29	48.53 ± 2.47	51.32 ± 9.78	44.22 ± 2.32	160.71 ± 11.03	120.24 ± 1.14	82.61 ± 2.51	34.37 ± 3.37	30.39 ± 1.61
	P. chicorii ITEM 17296	49.89 ± 9.11	7.92 ± 3.08	0.00 ± 0.00	0.00 ± 0.00	0.00 ± 0.00	65.02 ± 8.98	11.26 ± 0.74	0.00 ± 0.00	0.00 ± 0.00	0.00 ± 0.00	190.6 ± 3.6	104.36 ± 4.8	48.82 ± 2.18	53.11 ± 6.11	32.12 ± 3.12	94.51 ± 3.51	55.62 ± 5.38	44.57 ± 6.43	26.12 ± 2.88	30.26 ± 2.74
Pathogens	*E. coli* *ATCC 8739*	629.13 ± 11.97	285.57 ± 58.17	141.37 ± 2.23	62 ± 19	34.34 ± 5.34	872.27 ± 9.23	406.17 ± 4.67	209.06 ± 7.36	84.48 ± 3.18	49.83 ± 8.83	612.1 ± 12.8	325.87 ± 6.73	214.49 ± 13.39	81.22 ± 6.78	38.01 ± 7.01	966.08 ± 35.02	541.47 ± 10.13	238.85 ± 10.35	134.09 ± 3.89	68.83 ± 2.17
	E. coli *ATCC 35401*	513.18 ± 15.82	213.52 ± 5.02	138.12 ± 19.12	59.24 ± 1.76	25.14 ± 5.86	475.49 ± 14.41	273.85 ± 12.35	171.59 ± 9.69	60.32 ± 10.68	37.62 ± 9.38	296.65 ± 8.65	266.49 ± 24.99	138.16 ± 8.16	96.19 ± 5.19	36.77 ± 3.77	669.79 ± 18.11	388 ± 13.1	143.38 ± 26.08	108.39 ± 11.51	49.01 ± 10.01
	L. monocytogenes *DSM 20600*	359.32 ± 28.08	142.12 ± 6.88	80.08 ± 4.82	19.1 ± 7.1	0.00 ± 0.00	742.76 ± 15.24	180.33 ± 6.67	98.01 ± 10.01	61.43 ± 4.57	34.35 ± 1.35	469.05 ± 21	349.34 ± 7.64	269.09 ± 19.77	90.28 ± 0.72	57.09 ± 15.21	679.57 ± 20.33	346 ± 4.6	268.74 ± 9.84	86.51 ± 3.49	40.02 ± 1.02
	P. aeruginosa *DSM 939*	48.82 ± 4.82	47.76 ± 1.24	0.00 ± 0.00	0.00 ± 0.00	0.00 ± 0.00	185.59 ± 13.41	127.49 ± 6.51	78.34 ± 7.34	42.99 ± 3.99	0.00 ± 0.00	52.03 ± 3.03	41.66 ± 12.34	0.00 ± 0.00	0.00 ± 0.00	0.00 ± 0.00	92.2 ± 3.2	78.08 ± 7.08	68.04 ± 9.04	0.00 ± 0.00	0.00 ± 0.00
	Sal. enterica *ATCC 13311*	654.37 ± 12.83	176.77 ± 20.67	71.15 ± 3.78	35.97 ± 2.96	0.00 ± 0.00	1048.11 ± 49.71	207.86 ± 8.66	113.98 ± 26.05	75.66 ± 5.64	29.46 ± 1.81	391.65 ± 10.76	197.87 ± 2.73	52.48 ± 11.52	69.29 ± 8.29	23.4 ± 3.8	739.72 ± 31.34	355.72 ± 31.88	199.66 ± 11.45	141.76 ± 1.68	50.17 ± 7.83
	Sta. aureus *DSM 799*	2005.25 ± 100.62	402.59 ± 8.01	403.28 ± 4.24	327.1 ± 25.2	294.13 ± 71.23	4040.24 ± 49.84	2955.29 ± 107.64	517.95 ± 40.79	47.27 ± 14.26	5.9 ± 2.1	4819.24 ± 96.13	3217.84 ± 143.48	1241.78 ± 154.34	126.45 ± 63.23	56.49 ± 1.51	5221.42 ± 203.92	4484.97 ± 299.38	903.02 ± 18.44	90.78 ± 9.48	52.77 ± 15.12
